# Natural Savannah Systems Within the “One Welfare” Approach: Part 1—Good Farmers’ Perspectives, Environmental Challenges and Opportunities

**DOI:** 10.3390/ani15050677

**Published:** 2025-02-26

**Authors:** Marlyn H. Romero, Sergio A. Gallego-Polania, Jorge A. Sanchez

**Affiliations:** 1 Department of Animal Health, Faculty of Agrarian and Animal Sciences, Universidad de Caldas, Manizales 170004, Colombia; jorge.sanchez@ucaldas.edu.co; 2 Veterinary Science Research Group, Faculty of Agrarian and Animal Sciences, Universidad de Caldas, Manizales 170004, Colombia; sergio.gallego25728@ucaldas.edu.co

**Keywords:** tropical savannas, one welfare, human well-being, ecosystem services

## Abstract

The Colombian Orinoquia is considered one of the eight most important ecosystems in the world. Savannah ecosystems play an essential role in providing ecosystem services. The objectives were (a) to explore and identify the perceptions of traditional farmers and institutional representatives regarding human well-being, animal welfare and the environment, and (b) to identify environmental challenges and opportunities for improvement based on the “One Welfare” concept. Three focal groups were developed: male and female farmers and institutional representatives *(n =* 24) from Vichada. A thematic analysis, coding with an inductive approach and the definition of themes and sub-themes were carried out. The concept of being “a good farmer” explains the behavior of the producers, where the perception of human well-being is associated with the value of the family and the sense of pride in their tradition as “llanero”. Animal welfare was considered a symbol of profitability and prestige for cattle farmers, a concept that, for them, mixed traditional management, water supply, shade and feed, good health and the natural behavior of cattle. Regarding the environment, this concept is based on their awareness of the importance of conserving the natural savannah. Among the environmental challenges described are climate change, solid waste management and the use of controlled burns. Regarding opportunities for improvement, they proposed strengthening institutional dynamics, partnerships, environmental governance and education. Our results can provide information on the “One Welfare” approach and the motivation of farmers to care for animals and the environment, a fundamental aspect for developing effective intervention strategies.

## Simple Summary

The Colombian Orinoquia is an ecosystem of major importance due to its hydrological and biological richness. However, these natural landscapes share space and land use with livestock production systems. This study was developed to identify the perceptions and experiences of traditional livestock producers and institutional representatives on human welfare, animal welfare and the environment and to identify environmental challenges and opportunities for improvement within the One Welfare concept. Focus groups were held with producers (men and women) and institutional representatives from Vichada. Cultural and identity dynamics define the concept of human welfare. Animal welfare was based on favoring the animals’ natural behavior, maintaining the animal’s physical health and good care. The environment was focused on the conservation of the natural savannah. Climate change, solid waste management and the use of controlled burns were major challenges. As solutions, it was proposed that institutional work, partnerships, environmental governance and education should be promoted.

## 1. Introduction

Natural savannahs are tropical ecosystems spread over large regions of Africa, Asia, Australia and South America, covering approximately 15% of the Earth’s land surface [1,2]. Savannah ecosystems play an essential role in the provision of ecosystem services, such as biodiversity, water supply and regulation, groundwater replenishment, regulation of soil erosion, carbon stock and climate change mitigation, among others, as well as strengthening socioeconomic and cultural values [2]. Colombia has a total of 18,000,000 hectares of natural savannahs, of which 90% (16,000,000 ha) are located in the Orinoquia region, forming part of one of the eight most important ecosystems of the world, due to its biological and hydrological richness, which is home to around 17,420 plant species, 1300 bird species, more than 1000 fish species, 250 mammal species and 119 reptile species [3]. However, these landscapes and the natural dynamics of their ecosystems share space and land use with livestock production systems (mainly cattle farmers), agriculture and hydrocarbon exploitation [4]. This region has 1.7 million inhabitants from diverse cultures, settlers and indigenous people (“llaneros”), dedicated to the production of commercial crossbred breeding zebu cattle (5.1 million animals), which graze on some 9.4 million ha of natural savannahs in non-cultivable areas [5]. The term “llanero” refers to the ethnic group that emerged from the crossbreeding of the Arawaks, Andalusians, Canarians, and, to a lesser extent, the slaves brought to Colombia and Venezuela by the Spanish conquerors. In Colombia, they settled in the Eastern Plains or Orinoquia region, which includes the departments of Meta, Vichada, Casanare and Arauca. The term “llanero” implies a tradition or culture revolving around cattle ranching and working on horseback, an aspect that has given the region its identity [6].

Global recognition of the savannahs provides an opportunity to investigate local responses in the management of natural savannahs to foster multiple ecosystem benefits to sustain peoples’ livelihoods [2]. However, in these ecosystems, there is a dichotomy related to the achievement of sustainable development goals, in terms of addressing climate change, and on the other hand, meeting the demand for food of animal origin by intensifying land use and animal production, to ensure food security, nutritional health and the social sustainability of the population [7]. Likewise, the intensification of animal production has increased public awareness of conservation, health and environmental welfare, with the aim of promoting food security and sustainable food production [8]. In this context, it is paramount to analyze this duality with the “One Welfare” approach, to foster the integration of direct and indirect links between human welfare, animal welfare and animal production systems towards the environment, within an ethos where global challenges require this holistic approach [9].

The term “One Welfare” was first used in 2015 with the aim of promoting interdisciplinary collaboration for the international improvement of human and animal welfare, during which a website was created [10]. Its promoters based it on the “One Health” approach, a concept proposed over a century ago, which has been taken up by the World Health Organization [11]. However, although both approaches have common overlapping objectives and guidelines, the academic interests of the “One Health” concept have focused almost exclusively on the prevention and control of zoonoses, their impact on human health and the associated risk factors. In contrast, the “One Welfare” concept is based on ethical concepts of welfare focusing on the promotion of food security, the reduction of human and animal suffering, the study of social and cultural connections and the improvement of production practices within the livestock sector through the assessment and understanding of the value of high animal welfare standards in production systems [9,12]. Given that it is still a concept under development, research in this area is scarce. Some studies have reported the relationship between livestock ownership and the improvement in quality of life indicators for poor rural households in Southern Africa [13], the influence of the ideals and identity of the “good farmer” and their interaction with actions related to animal welfare [14], the connections between human and animal welfare [15], management of environmental issues, and benefits for the livelihoods of rural communities [2], among others. However, few studies simultaneously assess all three aspects.

Additionally, it is important to understand the sociocultural factors that mediate the relationship between farmers and animals, because animal care is part of an identity and has social and symbolic meaning for farmers [16]. The concept of the “good farmer” has been used scientifically in rural studies and social sciences to understand the attitudes, behavior and decision-making processes of farmers, both individually and collectively, as a result of their cultural processes, social norms, identity, role construction and feelings of responsibility, among other aspects. It also helps to understand and evaluate the conservation versus production identities of livestock farmers [16,17]. When farmers use this term, they primarily refer to those farmers whose practices demonstrate a “good” level of competence, which relates to their positive skills and emphasizes the ethical aspect of their farming practices [18]. These studies have described how individual values influence what a farmer considers a “good” or “bad” practice, based on their experiences and concerns related to their profession [14,19]. The concept of the “good farmer” is associated with their symbolic perception of skilled performance and visual representations of their practices [20], with the norms and culture of animal husbandry; therefore, sociocultural factors may influence their actions, behaviors and willingness to adopt programs established to enhance farm animal welfare [21]. Likewise, it is necessary to understand the communities’ perception of environmental factors affecting human and animal welfare, because producers are the ones who have the best knowledge of their area’s biogeographic characteristics and social conditions [22], which is useful information to develop strategies to adequately manage savannah ecosystems.

The use of qualitative research methods to understand human motivation, which is essential for developing effective intervention strategies, is currently on the rise [23]. Recent studies have used methodological strategies such as personal interviews and focus group discussions to understand the outlook of farmers, veterinarians and livestock managers on the motivations and perceptions of different aspects of human welfare, animal welfare and the environment in natural savannahs [2,24,25]. Qualitative research can also be exploratory and collaborative through the use of multi-stakeholder participatory approaches, which have been advocated as a way to develop more sustainable and concrete solutions to animal care challenges. Studies using the “One Welfare” approach are scarce in natural savannahs; therefore, the objectives of this study were (a) to explore and identify the perceptions and experiences of traditional livestock keepers and institutional representatives on human welfare, animal welfare and the environment and (b) to identify environmental challenges and opportunities for improvement within the “One Welfare” concept.

## 2. Materials and Methods

### 2.1. Ethical Considerations

This research was reviewed and approved by the Ethics and Animal Experimentation Committee of the Faculty of Agricultural Sciences of the University of Caldas—Activities with minimal risk (Act 1 August 2022) and informed consent was obtained for participation in the three focus groups. The participants were informed about the objectives, methods and implications of the study for them and the field of study; likewise, the participants were made aware of their right to choose not to answer any questions.

### 2.2. General Description

This study is part of a broader project aimed at “Developing a proposal for a model that integrates human welfare, animal welfare and environmental indicators to evaluate cattle breeding production systems in natural savannah, with a One-Welfare concept”. The results presented in this article are focused on applying qualitative participation strategies aimed at understanding the perceptions of the participants and the cattle raising practices in the natural savannah, which are based on traditional cattle raising. Although the term “a good farmer” was not used as such, within the set of guiding questions of the focus groups, the ideals of a good farmer emerged within the descriptions and justifications that guided the actions of the participants related to the “One Welfare” approach and how this relates to their traditional practices [18].

### 2.3. Area of Study

**Figure 1:**
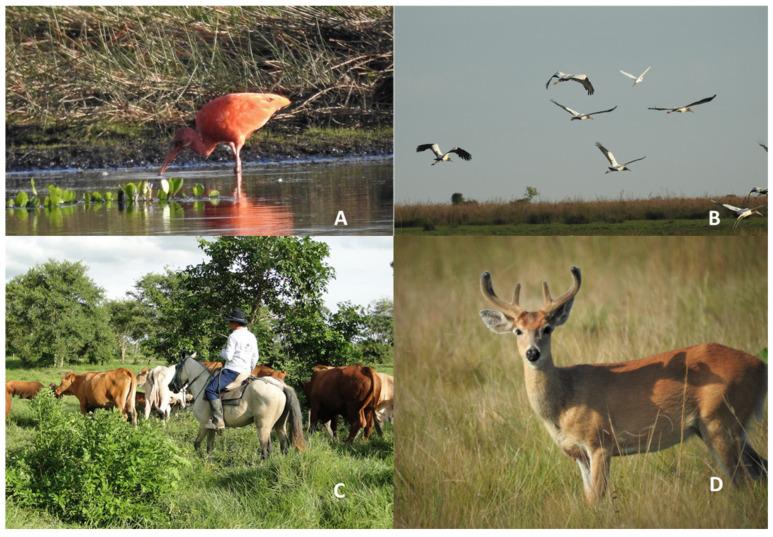
Natural savannah of the Colombian Orinoquia, La Primavera, Vichada. (**A**) Ibis escarlata or Corocora roja (*Eudocimus ruber*), (**B**) Gabán huesito (*Mycteria americana*), (**C**) “Llanero”, livestock and natural savanna and (**D**) Venado sabanero (*Oclocoileus virginianus*).

The savannahs of the Colombian Orinoquia present mosaics of forests and palm groves crossed by large rivers and streams that supply water to the great Orinoco River Basin. It is characterized by a large variety of grasses and legumes (more than 60 identified species). The department of Vichada is one of the largest in area in the country, with a total area of 10,018,540 hectares, 51% of which corresponds to agricultural areas, 46.5% to natural forests and the remainder to other land uses and coverage [26]. The production system is traditional extensive livestock farming, with low technological adoption and conventional production models [27]. Dual-purpose livestock (meat and milk) represents 15.3%, and meat production accounts for 84.7%. This region is strategic for supplying weaned cattle to the central part of the country, where the calves are kept with their mothers until the age of nine months. In Vichada, there is only one authorized slaughterhouse, located in the municipality of Puerto Carreño. During the summer, animals must be transported by land (on foot or in trucks) or by fluvial transport [26]. This study was conducted with breeding cattle producers in extensive grazing production systems in the eastern plains of Colombia (Colombian Orinoco), in the Department of Vichada, municipality of La Primavera (5°29′26″ N 70°24′33″ O), which has a total of 657 farms and an index of 141,228 cattle, whose livestock corresponds to 55% of the cattle and an area that represents 20% of the Department of Vichada [28]. The traditional use of Colombia’s natural savannahs revolves around Brahman cattle *(Bos indicus*) and their crossbreeds that are based on native grasses (*Axonopus purpussi* and *Schizachyrium microstachyum*) [5] (Figure 1, Figure S1 and Figure S2, and Video S1: Cattle drive).

### 2.4. Participants

The Participants were recruited intentionally (convenience sampling) with the help of representatives of the institutions responsible for animal health and welfare (Colombian Federation of Livestock producers-FEDEGAN, Livestock farmers’ committee of La Primavera, Livestock farmers’ committee of Vichada, La Primavera City Hall, La Primavera Department of Agriculture, Community Action Board) in the municipality of La Primavera [14,29]. The inclusion criteria for participation in the focus groups were (a) people ≥18 years old; (b) living in the municipality for at least the last five years, (c) being livestock farmers (men and women) of zebu cattle farms in natural savannah or being linked to state entities responsible for animal health and welfare.

### 2.5. Focus Groups

Three face-to-face focus groups (a group of male farmers, a group of female farmers and representatives of institutions) were conducted in the municipality of La Primavera on 12 and 13 November 2023, and were moderated by a sociologist with a Master’s degree in Social Sciences, together with the first and third authors, who were present taking field notes. The focus groups lasted 2 h each and were attended by 24 people, who completed a brief questionnaire about their personal information before starting the activity (Table 1). All the focus groups were recorded in audio and video and transcribed in audio by a professional transcriber (See Guide S1: Focus group).

### 2.6. Data Analysis

Thematic analysis was used to identify, analyze and report patterns within the written data using NVivo software (version 10.2.2; QSR International, Burlington, MA, USA). The focus group dialogues were professionally transcribed, and the transcripts were compared to the original audio files to ensure fidelity. The first author (MHR) and the sociologist read the transcripts and conducted initial coding using a bottom-up inductive approach aimed at identifying potential themes and subthemes from codes assigned to elements of the text that responded to the research objectives. Subsequently, the authors and the sociologist discussed the codebook, themes and sub-themes. Adjustments were made until a consensus was reached. An anonymous identification was made to each participant and codes were assigned to the quotes to illustrate key features of the themes and sub-themes. Quotes were selected to represent examples of a particular category within each theme; statements that reflected many responses and most clearly expressed a particular concept were specifically identified.

### 2.7. Positionality and Reflexivity Statement

We recognize that the positionality of the researcher affects how data are generated and analyzed [30]. The research team was familiar with cattle management in pasture and natural savannah conditions and included authors with expertise in thematic analysis and advanced studies in epidemiology, animal welfare and veterinary medicine. The first author (MHR) is a veterinary physician, professor and postdoctoral researcher with experience in conducting quantitative and qualitative studies to assess the welfare of production animals (cattle and swine) under commercial on-farm conditions and preslaughter. The second author (SAGP) knew all the focus group participants, as a PhD student living within the community for more than five years. However, he did not participate in the discussion sessions to control for possible bias. The first author (MHR) led the data analysis and writing of the article.

### 2.8. Data Presentation

**Figure 2:**
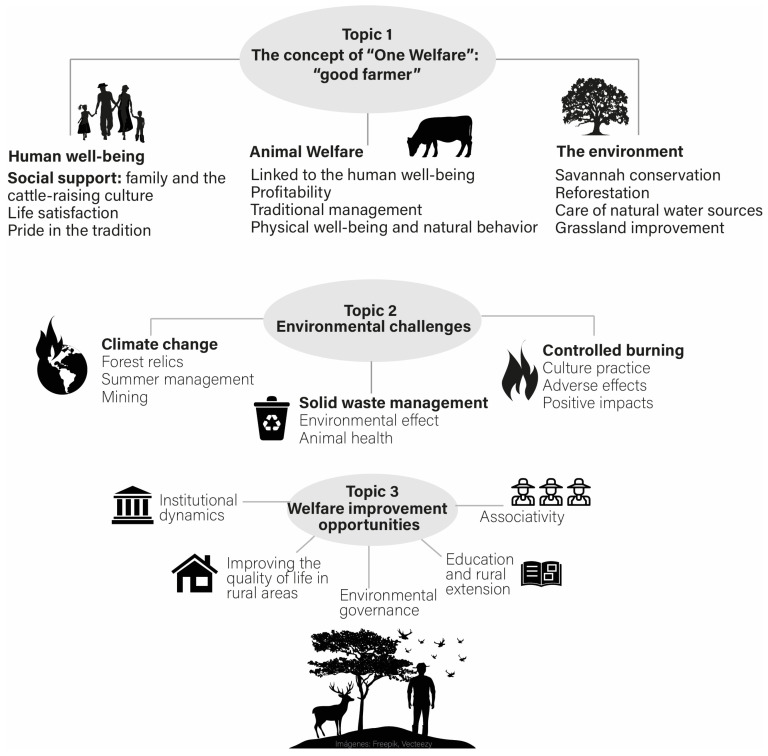
Thematic map of the analysis of three focus groups of producers and representatives of state entities on perceptions about the concept of “one welfare”, environmental challenges and opportunities for improving cattle farmer in natural savannah, La Primavera, Vichada.

A thematic map (Figure 2) was produced, and textual quotations were reported as examples of themes and identified by participant numbers. Clarifying text is included in parentheses; brackets were used when a word was changed to avoid using slang language or to preserve the identity of the participant and ellipses were used to indicate a part of the quote that was removed for brevity and did not change meaning.

## 3. Results

### 3.1. Demographics

The focus groups were represented by 10 female farmers (41.7%), 8 male farmers (33.3%) and 6 professionals from institutions (25.0%). Most of the group had low schooling and extensive experience in their activity, ranging from 20 to 71 years (Table 1).

### 3.2. Key Themes Identified

In general terms, socio-cultural factors define the three fundamental pillars that characterize the concept of “one welfare”: human welfare, animal welfare and environment, which are described in theme 1 and which are related to factors relating to farmer culture and pride in the tradition of the “good farmer”. The concept of human welfare is related to the value of the family, the producer’s satisfaction with life or his trade and pride in his tradition; animal welfare is conceived as an aspect that is linked to the profitability of cattle raising, traditional management that is passed down from generation to generation, the physical condition and natural behavior of the environment with the desire to conserve the natural savannah. Theme 2 presents the environmental challenges that affect human and animal welfare: (a) climate change, (b) solid waste management and (c) controlled burns. In theme 3, the following are proposed as opportunities for improvement: (a) institutional dynamics; (b) partnerships; (c) environmental governance and (d) education.

#### 3.2.1. Theme 1: Socio-Cultural Factors Defining Human, Animal and Environmental Welfare “One Welfare”

In the focus groups, male and female producers were guided to describe what aspects made them feel happy to be cattle farmers, as well as their perception of animal and environmental welfare. The factors that stood out in each of these components are described below.

##### Human Well-Being

- The family is the center of social unity in cattle-raising activities

For traditional livestock producers in natural savannah, the main factor of human well-being within the activity is to be in harmony with the family and keep it together: [P19_GR1M]: “For the llanero, the family is the basis of the home and of life, so it is necessary to keep it together, united” […] It is about thinking about how the work of livestock farming itself helps the family, neighbors and the community to evolve as a whole as a society. Our parents forged this land and its grandeur […] I hope that my children will continue to do the same”.

The cattle-raising culture in natural savannah is not unchangeable; on the contrary, it is permanently dynamic through complex economic and social processes that are transforming certain practices, perceptions and roles within the activity. The role of women in cattle raising is part of a factor of change with significant consequences for the practice of cattle raising in natural savannah in Colombia, as expressed by [P27_GR1M]: “The role of women is very important […] So the well-being of the family is based on the family union, even if they do not go into the corral […], but the emotional support and balance they give to the home is very valuable, the presence of women makes things very different”. Gender cultural transitions, generational change and the cultural evolution of livestock farming will not be discussed in this article, as they involve complex topics that deserve to be addressed in depth in an additional article, which is currently under evaluation for publication. This article will discuss aspects related to the sociocultural conditions of livestock farmers and the gender perspective (empowerment of women, changes in productive roles, inheritance, wage disparities and lack of recognition of women’s work), among other aspects.

b. Life satisfaction

When addressing the aspects that made the participants in the focus groups feel pride and satisfaction with their lives, factors related to cultural and identity issues stood out. The work of cattle raising is used to support the family, the region and society, a tradition that they want their children to continue. The participants selected farmer and the harshness of rural life as the factors that make them happy in their work, which has led them to value and “love their land”: [P37_GR1M] and [P45_GR2W] and [P38_GR1M]: “What makes me happy to be a farmer is my family and pride in raising livestock, the relationship between people and animals”.

c. Pride in tradition of the “good farmer”

The concept of the “good farmer” explains behavior as a result of cultural processes and producer identity. Livestock care skills are an important symbol of being a good farmer, where the love or passion for being a farmer arises from the specific knowledge of the activity, which is fundamentally based on the care, protection and sustenance of the animals. The focus groups highlighted that it is a tradition that defines livestock practice as a trade, where knowledge is inherited and transmitted from generation to generation, also transmitting certain roles or social roles of men and women within specific activities, where the woman takes care of the calves and the rougher work is done by the man, an aspect that coincides with what was described by the farmers [P2_GR2W]: “since I was a child I have been raised on a farm, handling cattle and when I was a girl I had to help take care of all the small calves and take them away from the cow to be milked” and [P10_GR1M]: “I am a man who has had cattle or I have been in contact with cattle since I was a child”.

The identifying characteristics of the “llanero” are maintained as part of the subjectivity of cattle management in the natural savannah. There are some symbolisms associated in a practical way with livestock activities that are maintained as a need to strengthen regional identity, such as the use of hats, clothing, work tools and the use of singing to handle cattle, symbols that are part of the cultural roots defined as “being a llanero”. For livestock management, producers use “singing” as a special form of bonding between the llanero and their animals, as stated by the farmer [P10_GR1M]: “The cattle like it when you sing to them, whistle to them, when you are with them, so the cattle are relaxed”.

Singing is considered a natural gift that is transmitted from generation to generation and is developed in a particular way by each farmer. The “llanero song” is considered an intangible cultural heritage of humanity and is performed for various purposes, such as calming the animals, strengthening bonds with them, remembering oral traditions, paying homage to the countryside and work and accompanying routines with the livestock. Work songs are part of the Llanero tradition during milking. The herding song accompanies the work routines on the plains (Video S2: Herding song; Video S3: Milking song). The moderator inquired about the possibility that this cultural practice could be taught in a university, for which the cattlemen (men and women) indicated that it was something innate and specific to their culture, as highlighted by the cattlemen [P16_GR1M]: “Not at all. You don’t learn that not even by watching. It is something natural for each person, which has been learned by working in the plains since childhood. So singing is a gift […] It is cultural. I know people who drive cattle up and down, but they don’t have that gift” and [P1_GR2W]: “Since childhood I learned to call the cows. In my farm, there was one that was called Cafetera and I would shout: “Cafetera, come on old lady” (singing, whistling), and she would poke her head out (mooing)”.

##### Animal Welfare

- Link with human welfare and the profitability of livestock farming

Animal care is part of the identity of livestock farmers and has a socio-symbolic value in livestock farming communities. For producers, good cattle raising means profit, prestige and success. It is estimated that the living conditions of families, managers and animal handlers are determinants of animal welfare and human welfare, according to the living conditions in the natural savannah. The farmers in this study, despite having low schooling, recognize from their life experience and empirical knowledge the needs of cattle and their close relationship with human welfare, as expressed by the producer [P11_GR1M]: “Animal welfare must always be compared with the welfare of people. If we like to drink water of a certain quality […], so do the cattle and in sufficient quantity. We like to sleep well, […] and the same happens with animals and we have to adapt to their needs. Animals feel the same way we feel, they think, they feel fear and they recognize people. So, all these things must be taken into account in animal welfare in order to understand it.

b. Traditional management

The relationship between humans and animals depends on some human characteristics, such as the person’s familiarity with the animal, attitudes, skills and knowledge. Savannah cattle farmer is transmitted generationally, as expressed by the farmer [P5_GR2M]: “… My link with cattle farmer also comes from the family, from heritage, from tradition. […] as life goes by, you see how your father goes and handles the cattle, goes to work the plains, then you get a taste for cattle raising”.

“Coleo” is a cultural action to demonstrate the proper interaction between man and animal in terms of strength and dominion over the task of “being a good farmer” and particularly “llanero”. “Coleo” is a traditional sport that originated during the process of branding calves in the corral. Two ranchers work as a team: one lassos the animal, while the other grabs the cow’s tail and brings it down. The one who lassoed the animal then brings the hot iron and brands the calf. This process is called la hierra (branding of cattle), and it is carried out every six months, in May and November. Later, “coleo” expanded to the savannah, where it is practiced from horseback to catch an animal that is getting away from the herd.

The regional rootedness of the practice of “coleo” faces processes of change that occur through a process of cultural conflict in the face of animal mistreatment, a conflict that has introduced a discussion of change or even abolition of this practice, about which women have been more receptive, as expressed by the cattle farmer [P30_GR2W]: “Well it makes me uneasy to see animals being mistreated. The culture of the Llano makes them mistreat animals a lot, for example, in “coleo”, that is why I am not a fan of this sport, because it makes me feel sad when they mistreat animals”.

c. Physical well-being and natural behavior

Livestock handling skills are an important symbol of the “good farmer”, as well as the ability to take care of health problems, provide sufficient rest possibilities and quality and quantity of water and feed and encourage the natural behavior of the animals. In this study, the meaning of animal welfare for farmers consisted of understanding the animal in its conditions of freedom, protection against adverse weather conditions and health care: [P17_GR1M]: “Another factor of animal welfare is the assistance of a veterinary professional when surgery or treatment is required”. Farmers recognize the importance of accessibility to sufficient pasture, good quality water and mineralized salt supply: [P11_GR1M]: “Animal welfare is to make sure that the animals have pasture and water, and that it is clean. Farmers always opt for making wells in the savannah so that all cattle can drink water and also have their mineralized salt”; likewise, they consider thermal comfort important: [P09_GR1M]: “Animal welfare consists of improving pastures, they need shade.’ […] but if we have cattle and do not provide them with shade, the animal has to put up with too much heat”.

##### Environment

- Savannah conservation

Cattle farmers are aware of the great importance of the savannah and the concept of preserving and improving the environment has been on the rise, favoring the implementation of silvopastoral practices as a new approach to conservation and the planting of native species. Likewise, women express more awareness of conservation, burn control and traditional pasture and forage management. Women are receptive to these issues of change, as expressed by the cattle farmer [P14_GR2W]: “We consider that the environmental issue is fundamental. Because of our culture, we did not realize how important environmental concerns were, but today with global warming, what we need most is to implement a silvopastoral culture […] The animals also feel the sun and the heat; as women, we are more inclined to plant the bushes or then trees, because a pasture requires shade”.

The farms in the savannah do not have fences to mark the boundaries of private property and respect the natural boundaries established by neighbors, because they consider them to be more environmentally friendly, as expressed by farmers and members of state institutions [P7_GR3I]: “… to put concrete posts, it is a matter of environmental welfare so that trees are not cut down. […] a wooden fence is better, if a cow knocks it down it is useful for firewood, or if it is a tree it gives shade to the cattle, but a concrete post isn’t useful”.

Likewise, the link between animal welfare and environmental issues is recognized, [P22_GR2M]: “[…] some of you mentioned that it is important to maintain certain conditions on the farm where water management is adequate and sufficient for the animals, with proper management and control of pastures and tree planting, but these conditions have to do with both animal welfare and environmental issues”.

The farmers respect the knowledge received from their predecessors and wish to conserve their environmental management practices, as expressed by the institutional representative [P37_GR3I] in the focus group: “the farmers here know a lot about caring for the environment, for example, they know which moriches [native forest reserves] conserve water, they know how to manage them and at what time the animals should enter”. The region is rich in river tributaries and the farmers recognize their importance, although they did not speak clearly about how they conserve them, they did express their importance, which is why they consider their cattle raising to be sustainable.

#### 3.2.2. Theme 2: Environmental Challenges Affecting Human and Animal Welfare

- Climate change

Farmers are aware of climate change and the importance of tree planting, pasture improvement and water and forest management, as well as the relationship of climate change on human welfare, animal welfare and the environment. Climate change is considered as a factor that has influenced the disappearance of savannah farms, as indicated by the cattle farmer [P22_GR2M]: “[…] before they worked farms for 60 days and still didn’t get around all the Llano. But cattle raising has also been disappearing, not only because of social problems [armed conflict], but also because of climate change”. However, as it is a new program in the region, the representatives of the institutions consider that it requires an integrated approach between institutions, farmers and the state, and therefore, a lot of effort and training, as expressed by [P6_GR3I]: “You analyze the issue of climate change and everyone chooses their own way. This is also the case with other environmental services issues”.

In the Orinoco region, the rainfall pattern is bimodal with very marked climatic conditions, with a very intense summer that drastically affects food security and animal welfare, as indicated by the farmer [P9_GR1M]: “One of the difficulties we have in the savannah is the high temperatures, not only because of the heat but also because the grass dries out a lot and since it does not rain, the grass does not grow and the animals die of hunger and we have no water or crops to eat”.

One of the aspects they were concerned about is the management given to the natural savannah by the indigenous reserves, due to their extractive culture and lack of environmental conservation, factors that contribute to climate change, as stated by the institutional representative [P14_GR3I]: “the indigenous people have an extractive mentality. The state has developed food security programs and the adoption of sustainable systems, such as solar power systems, but they do not use them, they sell them. […], the same goes for wildlife hunting”. Another challenge is the threat of oil exploration in the region, which has not yet occurred in the department of Vichada, but which the focus group participants have observed in their neighboring areas, with negative effects on the environment: [P25_GR3]: “we still have a pending environmental issue and that is the threat of oil exploration and exploitation because they will surely arrive at some point, as in Caño limón, Cusiana and Rubiales”.

b. Solid waste management and disposal

Due to the high scattered nature of the farms, there are no household waste collection systems in the natural savannah, and solid waste is disposed of by burying and/or burning. Organic waste is used as fertilizer for crops, but there is concern about the handling and management of plastic, due to the contaminating effects on the soil and the risk of consumption by cattle, with adverse effects on animal health (tympanism and death), as expressed by [P28_GR2M]: “On the farms, garbage is burned because there is nothing else to do; some bury it in a hole. Organic waste is used as fertilizer for crops, but plastic bags and other waste, as there is no garbage collection, are burned. Glass is also buried”.

c. Controlled burning

Burn management is a frequent practice applied in many tropical savannahs to promote the vigorous regrowth of natural vegetation for cattle grazing. The management and control of burns has been a traditional practice and part of the cultural knowledge of the territory. These aspects do not appear as an environmental concern as long as they are done in the traditional way, which is considered to be correct. There is concern because some farmers do not burn based on the knowledge of the savannah and forget the importance of preserving wetlands, forests, fauna and flora, as expressed by [P36_GR1M]: “… the savannah itself is in charge of recovering. There are people who burn and burn […], but nature has its own control. There are people who burn in a controlled way, with experience […], but there are others who do it in an uncontrolled way, really, we must try to be very careful with wetlands because they are ecosystems that must be taken care of”.

#### 3.2.3. Theme 3: Welfare Improvement Opportunities with a “One Welfare” Vision

There is concern on the part of livestock farmers and representatives of institutions about the most sensitive problems affecting the welfare of the natural savannah, which are briefly presented below: [P18_GR1M]: “There is another thing that is kind of contradictory, for example, burning is done to improve the pasture […], but it ends up deteriorating the environment with burning […] which finally, can also harm animal welfare”.

- Institutional dynamics

The possibility of extending channels of communication between institutions and professional farmers produces formative interactions with a professional and technical influence on management and land use practices in the cattle raising activity, contributing a factor of change-oriented to technical and scientific rationality. Each institution sets its own objectives and goals, but there is not always leadership from agents that promote integration in order to provide a comprehensive support service to farmers. Institutional action raises good intentions that often depend on non-institutional factors, such as the state of the roads or the conditions or distance to the cattle reception centers for slaughter. These are variable elements beyond control in a large territory with deficient roads and transportation services. The consensus of the participants is summarized in the following comment: [P5_GR3I]: “Incorporating a new culture requires support […] and we still need it. This is accompaniment by the state […]. If we aren’t given help with these technologies they want to incorporate, it is difficult for them to enter the territory and for us to change the “chip” of an age-old culture”.

Likewise, the participants considered that it is essential to promote land-use planning programs and use institutional information systems to establish improvement programs, as expressed by [P20_GR3I]: “There is no organization, land-use plans do not exist, they give us some graphs and a very nice system, but the institutions do not use it to create the programs”.

b. Associativity

External factors include the sector’s lack of capacity to build and maintain partnerships. Each person defends his or her activity in a very individual manner. To the extent that the results of technical and social accompaniment are collectively recognized, families become more receptive and integrated into the process of change. The “llanero” has the culture of working individually, but the younger generations are aware of the advantages that can be achieved by working as a team and as a syndicate, as expressed in the focus groups by the cattle farmer [P19_GR3I]: “… At this time there should be more associativity because opportunities are emerging, the country is changing, needs are changing, market dynamics. […], and it is time to work as a team”.

c. Improving the quality of life in rural areas.

There is a need to improve living conditions in rural cattle raising areas, especially in terms of health services for the population in the more remote areas. The centralist model delays interventions and security problems prevail, such as cattle rustling and public order problems led by some indigenous groups that hinder access to the region through strikes and toll collections, as expressed by the cattle farmer [P26_GR3I]: “There is an issue that is difficult to deal with, which is the indigenous element. […], you go to many communities here and they put tolls, because of the issue of the indigenous reserves and the management of the territories. […] and it is not a question of driving them away, but to force them to preserve this territory, because they are the ones who are most knowledgeable and are the ones who are in the area”.

Another aspect that requires government intervention is road construction, water and sewage services, solid waste collection, and other public services. Access to the natural savannah is by land or river, depending on the bimodal rainy season. Roads are not paved, and river transportation is expensive for the savannah’s inhabitants, but it is the most commonly used means of transporting food, goods, raw materials, inputs and animals, among others. In the case of the mobilization of fattening cattle, transportation includes these two routes, but is routinely done on foot, with journeys that last up to 8 days; however, cattle farmers consider that it is more viable and friendly to animal welfare, as expressed by [P34_GR1M]: “Transporting cattle on foot is better, because the animals lose more weight when they are loaded on a truck, than when they walk. The removal of cattle on foot takes a week, but they arrive in better condition. In a boat, for example, it is terrible”.

d. Environmental governance of the territory

Livestock governance needs to be strengthened to jointly address sustainability issues in the sector. The cattle farmers expressed the need to discuss support and accompaniment issues to improve this activity’s social conditions. Likewise, they consider that state investment is required following the taxes they must pay to practice their trade of ranching, as expressed by [P11_GR1M]: “Well, I think it is time that the government started to pay attention to us farmers, that they do not collect so many taxes from us for having cattle. The money could be invested to improve the grassland. The countryside alone does not produce anything, we the farmers are the ones who are producing”.

An example of an environmental governance strategy and the joint work between the state and the community, environmental education related to solid waste management has been initiated in the region, and the women farmers have been receptive to this initiative because they consider it very positive to preserve the savannah, as stated by the farmer [P28_GR2W]: “… The truth is that I value what this boy [environmentalist who has given a letter of introduction to the whole municipality in conservation] is doing. He has been educating this entire municipality on the culture of waste management, to classify waste, because here we have never had that habit, but that helps us in the environmental welfare issue and helps us to understand that it is a fundamental issue”.

e. Education

The inclusion of pedagogical forms or approaches that respond to the need to approach the rural population requires an institutional and state effort, according to those expressed by the participants in the discussion groups. According to their assessments, education programs are required starting with the teaching of the Llanera culture to children, adolescents and adults, to preserve the tradition and establish methodologies and improvements to enhance their standard of living, animal welfare and environmental care, as summarized in the comment [P8_GR3I]: “It is one thing to teach children and another to transmit the information to adults so that they change. It is difficult to explain to a 70-year-old man that you have to fence, that cows feel and take decisions. They are familiar with the information through tradition. […] there are some farmers or technical assistants who have made their professional lives here in Vichada, so it is easier for farmers to make simple changes such as fencing, dividing paddocks and rotation”.

## 4. Discussion

The assessment of human well-being is complex and focuses on a triarchic theoretical approach that considers hedonic well-being, eudaimonic well-being and social well-being. Hedonic or subjective well-being defines how people feel, i.e., their emotional experience of their quality of life [31]. Cattle farmers from a cultural and social point of view need a “social license” to operate and within this context, the ideals of the family and the role of women as the fundamental support of the family, form the central core of their life satisfaction [19], as expressed by the traditional cattle farmers in the current research. Likewise, they felt pride in their identity practices as “llaneros” and their special connection through singing and close management with cattle, expressed as a positive emotional state of the profession of being a “good farmer” [32]. On the other hand, eudaimonic well-being is associated with the processes of participation in meaningful activities, i.e., self-realization, positive relationships with others and personal growth [33]. Finally, social well-being reflects the evaluation of one’s own circumstances and the functioning of society [31]. These last two aspects were not assessed in this study and should be the subject of future research.

The traditional farmers in this study exhibited a set of idealized practices and principles as part of their identity, which are indicative of good animal welfare and seen as symbolic of being a “good farmer” [14], such as the singing used during interaction with animals, handling animals in pasture conditions that favor natural behavior, the inheritance of tradition and care for the land and animals, among other aspects. The “good farmer” identity integrates a set of shared cultural symbols, practices, principles and ideals to better understand why producers emphasize and view particular practices as ideals [18,20]. In the natural savannah of Vichada, producers associated animal welfare with the care of the animal’s physical body (good nutrition: supply of water and feed of good quality and quantity), care of the animal’s physical environment (protecting it from adverse weather conditions), management of its health and favoring its natural behavior, perceptions shared by different livestock farming cultures, as described by Vigors et al. [14] in the United Kingdom, Logstein and Bjørkhaug [19] in Norway, Romero et al. [34] in Colombia, in cattle fattening farms under pasture conditions and Martínez et al. [35] in certified swine productions. Likewise, farmers empirically recounted a framework of resources of “good life” opportunities for breeding cattle under natural conditions, within a context that is currently defined as positive welfare [36,37].

Good livestock farmers pay attention to the complexity of ecological systems and adopt a view of nature as an integral part of livestock production [20]. Among the environmental challenges, the farmers identified anthropogenic climate change, considered a threat to human and animal food security [38], which reduces the productive parameters of cattle, favors the spread of diseases, increases cattle mortality [39], lowers feed conversion [40] and impacts the quality of forage crops and forages, water availability and biodiversity [41], among other aspects. The livestock farmers in the present study were aware of the climate change issue with a “One Welfare” approach. In this context, livestock farmers demonstrated a harmonious and respectful attitude towards the environment, recognizing the need to conserve the natural savannah as a climate change mitigation measure, as well as implementing silvopastoral systems compatible with the natural ecosystem, the use of tree shade, the care of water sources, drought management and protecting animals against heat stress, as described by livestock farmers from Pakistan [42], Hong Kong [17] and Indonesia [2].

Cattle farmers are concerned about mining and energy activities that are already being developed in neighboring departments, as a post-conflict state policy to boost biofuel production to promote the country’s economic development, due to their potential to expand deforestation, soil and watershed contamination, as well as to favor climate change. However, Colombian regulations promote biodiversity conservation, the development of protected areas and farmer collective lands, among other aspects [43,44]. It is therefore required that the government adopt precautionary measures such as the development of zero deforestation value chain agreements and evaluate the impacts of this activity on the environment and the health of citizens [43]. With regard to environmental governance, Colombia has made progress in regulations on the legal protection of water, in response to the growing impacts of climate change and anthropogenic environmental destruction [45]. Likewise, ecosystem-based adaptation projects have been developed among local communities, in which participants highlighted increased climate change knowledge and awareness, gender empowerment, employment opportunities and perceptions of climate regulation [46]. However, in the present study and others conducted in Colombia, the rule of law is considered weak and there is a lack of state presence in rural communities, in terms of the provision of social, environmental and health services. Similarly, there is less law enforcement presence, and corruption at the local, regional and national levels could have an impact on the transparency of the institutions [47].

Throughout the savannah biome, ligneous vegetation is cleared to increase the productivity of herbaceous grasses. While clearing may result in increased semi-arid dystrophic savannah grassland production in the short term, it is uncertain whether productivity is maintained in the long term and how it affects soil nutrients and organic carbon stocks [48], as well as biodiversity conservation [49]. In this study, the cultural practice of clearing through traditional logging and burning was observed, a situation that has been the cause of debate in many articles, because some researchers define them as traditional practices used to prepare the land, facilitate the cultivation of crops and maintain the dynamics of the savannah [2]. However, other authors have shown that when burns are not carried out properly, forest fires frequently occur at the end of the dry season or during prolonged drought conditions [49]. These uncontrolled burns pollute the air with emissions that are harmful to human health and have negative effects on natural resources and biodiversity [2,50]. Additionally, they have the potential to alter soil carbon storage, but it is also known that fire improves biodiversity in native savannah [51], floristic composition and ecological processes resulting in richer and more diverse forests [25]. Other factors that increase the potential use of burns are related to the following: (a) burns favor the compositional mix of trees and grasses and avoid the transition to perennial shrubs, thus allowing the perpetuation of the savannah grassland matrix [2], and (b) fires warm the soil and reduce accumulated litter, allowing sunlight penetration and the creation of favorable conditions for the growth of new grasses and flowers and (c) are beneficial for maintaining the savannah structure and biodiversity [50,52]. We propose conducting studies under the conditions of the savannahs of the Orinoquia, in order to establish the environmental and social impacts, as well as to evaluate actions to carry out burning efficiently.

Among the opportunities for improvement, the need to strengthen institutional dynamics and promote education programs was identified. Education and extension services are key to informing smallholders and raising awareness about adapting to new environmental changes and adopting more sustainable production practices [38]. However, the intention is more about adaptation to innovation by producers, rather than the adoption of technologies that are not feasible in their circumstances [53]. Currently, the farmer-to-farmer participatory approach is being promoted, where the farmer is the main extension agent, but requires training, living with the local community, understanding the context and giving feedback on the process with their life experiences [54,55], an aspect that was advocated by the participants of the current study. Relationship values and local attachment strengthen farmer–farmer extension, relational values support the adaptive capacity of connected farming communities and strengthened relationships support the use of more appropriate agricultural innovations and support participatory behavior [54]. Paradigm shifts are gradual, and require actions that foster collaborative processes (social learning) that incorporate farmers’ perspectives in the development of knowledge and innovation, as well as their contribution to defining objectives, strategies and programs collaboratively [56].

Knowledge transfer is more effective if the potential user of the information (farmers) has confidence in the new knowledge, and is therefore willing to act on it. It is also necessary that, from their perspective, the new knowledge or innovation meets these three characteristics: credibility (trust), relevance (matching their needs) and legitimacy (based on their social and cultural context) [57]. The focus groups in this study expressed the positive impact of the solid waste management program being implemented in the region, led by an environmental engineer (who trained and returned to Vichada), which supports what other authors have described as the importance of connecting young and local people to education and extension programs, as well as linking women and other marginalized groups [58]. Another strategy that could be implemented is field schools that encourage consultative or collaborative participation at the farm level. This strategy has been used successfully in the climate change adaptation behavior of Kenyan farmers, with results showing that social learning improves the adoption of complex farming practices compared to traditional models [59].

Another aspect considered critical by livestock farmers was the improvement of rural livelihoods, an issue that has had an impact, for example, on the 17% decrease in the number of farms in Switzerland [60]. It has been proposed that policymakers, both at a local and national level, should improve resources and services, expand social networks and seek options to favor the commercialization of products to add value and foster rural resilience [61]. Associativity was considered as another factor of interest by the focus groups. Good producers can also be recognized for their involvement in local community activities and social practices such as being good neighbor [62]. Collaborative work is a participatory rural development strategy, which not only favors economic benefits (access to sound markets, more affordable commodity prices and provision of services) but also addresses environmental and social challenges (mutual aid) [63], fosters resilience to debt pressures and grant funding [64] and is conceived as a means to enhance women’s economic and social empowerment [65]. Factors that contribute to ensuring the continuity of collaborative work include (a) the attachment of the community to its land, (b) identification of a unifying cause, (c) the frequency and depth of social connections, (d) the resources, knowledge and skills that individuals bring to these groupings and (e) the fostering of cooperative values (trust, loyalty, democratic participation and solidarity) [64,66].

Finally, qualitative studies have limitations that may lead to information biases, which were controlled by the researchers and are outlined below: (a) Personal interests of the participants. In this case, the farmers may have emphasized subjective and non-negotiable positions (personal perspectives and beliefs that could be influenced by their context and interactions with others), which were clarified and interpreted through the methodological analysis conducted under the guidance of a sociologist expert, (b) controversies and shifts in language, which occur when dialogues veer toward irrelevant aspects of the study and (c) the interpretation of regional expressions or idioms unique to the group, biases that were controlled through expert moderation to understand the attitudinal and cultural meanings of the topics discussed. The validity and reliability of this study were ensured through investigator triangulation, an external review by the specialized advisor (sociologist) and the taking of reflective notes, audio recordings and transcripts [29].

## 5. Conclusions

The qualitative methodology of focus groups used in this research allowed us to promote dialogue, dynamic discussion and the contrast of opinions among participants, who shared and self-identified in the practice of traditional livestock farming. In addition, during their discussions on these topics, they developed in a complementary way “a type of knowledge” based on a variety of attitudes, experiences and beliefs, representing a fundamental empirical data set from a conversation between “peers” who identify territorially with a practice that needs to be understood socially and culturally. This allows for a more nuanced understanding of participants’ perspectives from their viewpoint as a “good farmer”. This research provides information on the norms, values, cultural aspects and attitudes of traditional livestock keepers in a natural savannah, giving meaning to the ‘One Welfare’ concept. This information is important for developing effective intervention strategies and opens new research opportunities.

The results of this study promote adjustments to policies and regulations regarding environmental management, offering alternatives to burning, such as techniques for conserving and restoring natural forests that contribute to biodiversity conservation and its benefits. Additionally, the adoption of new practices is encouraged to increase productivity and resilience against climate change and animal welfare. Similarly, the development of knowledge transfer programs that integrate local actors with academic training should be promoted, fostering animal welfare practices and sustainable livestock practices adapted to “llanera” conditions and culture. Research using mixed methods (both quantitative and qualitative) is needed, where qualitative research is used to explain and complement quantitative findings, including the study of comprehensive strategies to address challenges from a holistic “One Welfare” approach while integrating culturally binding interventions. Likewise, studies exploring social and eudaimonic welfare aspects are needed, as they can guide collective interventions and generate greater adherence to compliance with animal welfare and environmental legislation, with a participatory approach to policy formulation.

## Figures and Tables

**Table 1:** Demographic information of focus group participants (*n* = 24) on perceptions related to the One Welfare approach, La Primavera, Vichada.

Characteristic		Female Farmers *n* (%)	Male Farmers *n* (%)	Institutions *n* (%)
Gender	Male	“-”	8 (33.3)	6 (25)
	Female	10 (41.7)	“-”	“-”
Age	28–38	4 (16.7)	2 (8.3)	“-”
	39–49	3 (12.5)	3 (12.5)	3 (12.5)
	50–60	1 (4.2)	1 (4.2)	2 (8.3)
	61–71	2 (8.3)	2 (8.3)	1 (4.2)
Level of education	No education	1 (4.2)	3 (12.5)	0 (0)
	Primary	3 (12.5)	5 (20.8)	2 (8.3)
	Secondary	4 (16.7)	“-”	“-”
	Post-secondary	2 (8.3)	“-”	4 (16.7)
Years of experience	20–30	4 (16.7)	1 (4.2)	1 (4.2)
	31–41	3 (12.5)	3 (12.5)	2 (8.3)
	42–52	1 (4.2)	2 (8.3)	2 (8.3)
	53–71	2 (8.3)	2 (8.3)	1 (4.2)

## Data Availability

Data will be made available on request.

## Supplementary Materials

The following supporting information can be downloaded at https://www.mdpi.com/article/10.3390/ani15050677/s1, Guide S1: Focus group; Figure S1: Animals in savanna natural, Vichada, Colombia; Figure S2: Farmer-livestock interaction, Vichada, Colombia; Video S1: Cattle drive; Video S2: Herding song and Video S3: Milking song.
