# Supplementary material for: Natural Savannah Systems Within the “One Welfare” Approach: Part 1—Good Farmers’ Perspectives, Environmental Challenges and Opportunities
Source: Animals (Basel). 2025 Feb 26;15(5):677. doi: 10.3390/ani15050677 (PMC11898485; doi:10.3390/ani15050677)
Supplement: Supplementary file 1 [file animals-15-00677-s001.zip › Guide 1 Focus Group.pdf]

GUIDE FOCUS GROUP

General aim: to explore and identify the perceptions of traditional farmers and institutional representatives regarding human wellbeing, animal welfare and the environment, and b) to identify environmental challenges and opportunities for improvement based on the "One Welfare" concept.

Key questions for exploring the local farmer perceptions with savanna ecosystem based on the One welfare concept.

The "One welfare" concept based on the experiences and perspectives of farmers (men and women) and institutional representatives.

Q1. What is animal welfare for you?

Probing Question: Is there any connection between human wellbeing and animal welfare?

Q2. What factors make you happy to be a farmer (llanero)?

Q3. What lessons have you learned from ranching as a life experience?

Q4. What are the reasons that make you love cattle?

Probing Question: Do they use singing to manage cattle?

Probe: What practices characterize them as "llaneros"?

Q5. Have they received training in animal health and welfare from governmental institutions?

Q6. For you, what is environmental welfare?

Q7. Is there any relationship between human welfare, animal welfare and environmental welfare?

Environmental challenges with a "One welfare" approach.

Q8. What are the environmental factors or cultural practices affecting the natural savannah that impact human, animal and environmental welfare?

Probe: What are the reasons for burning: social, cultural, behavioral, economic, technical?

Probe: How is the management of solid waste in the savannah?

Probe: How often does drought occur in the savannah?

Q9. What are the clearing practices for the savannah areas dedicated to cattle ranching?

Q10. What are the current local policies on savannah management?

Probe: What factors of climate change, human, animal and environmental well-being?

Opportunities for improvement

Q11. What strategies do you propose to solve the problems identified in the natural savannah?

Probing Question: What role do government institutions play to improve animal health, human welfare and protect the environment?

Probing Question: Do you have producer partnerships in the region? Do they belong to them?

Q12. Do you have any additional comments on improvement strategies that we have not discussed?
