# Supplementary material for: Natural Savannah Systems Within the “One Welfare” Approach: Part 1—Good Farmers’ Perspectives, Environmental Challenges and Opportunities
Source: Animals (Basel). 2025 Feb 26;15(5):677. doi: 10.3390/ani15050677 (PMC11898485; doi:10.3390/ani15050677)
Supplement: Supplementary file 1 [file animals-15-00677-s001.zip › supplementary figures.pdf]

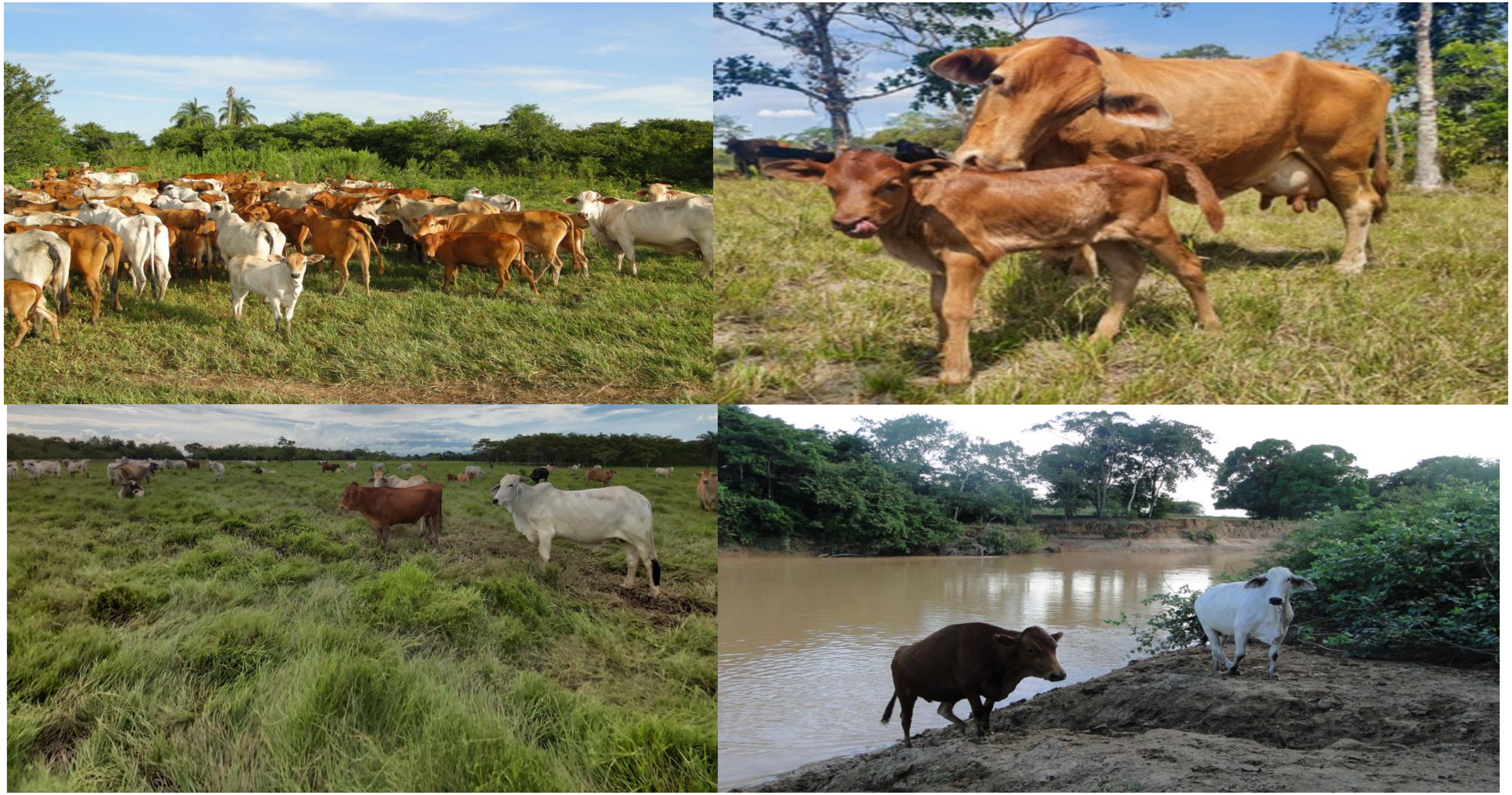

Figure S1. Animals in savanna natural, Vichada, Colombia

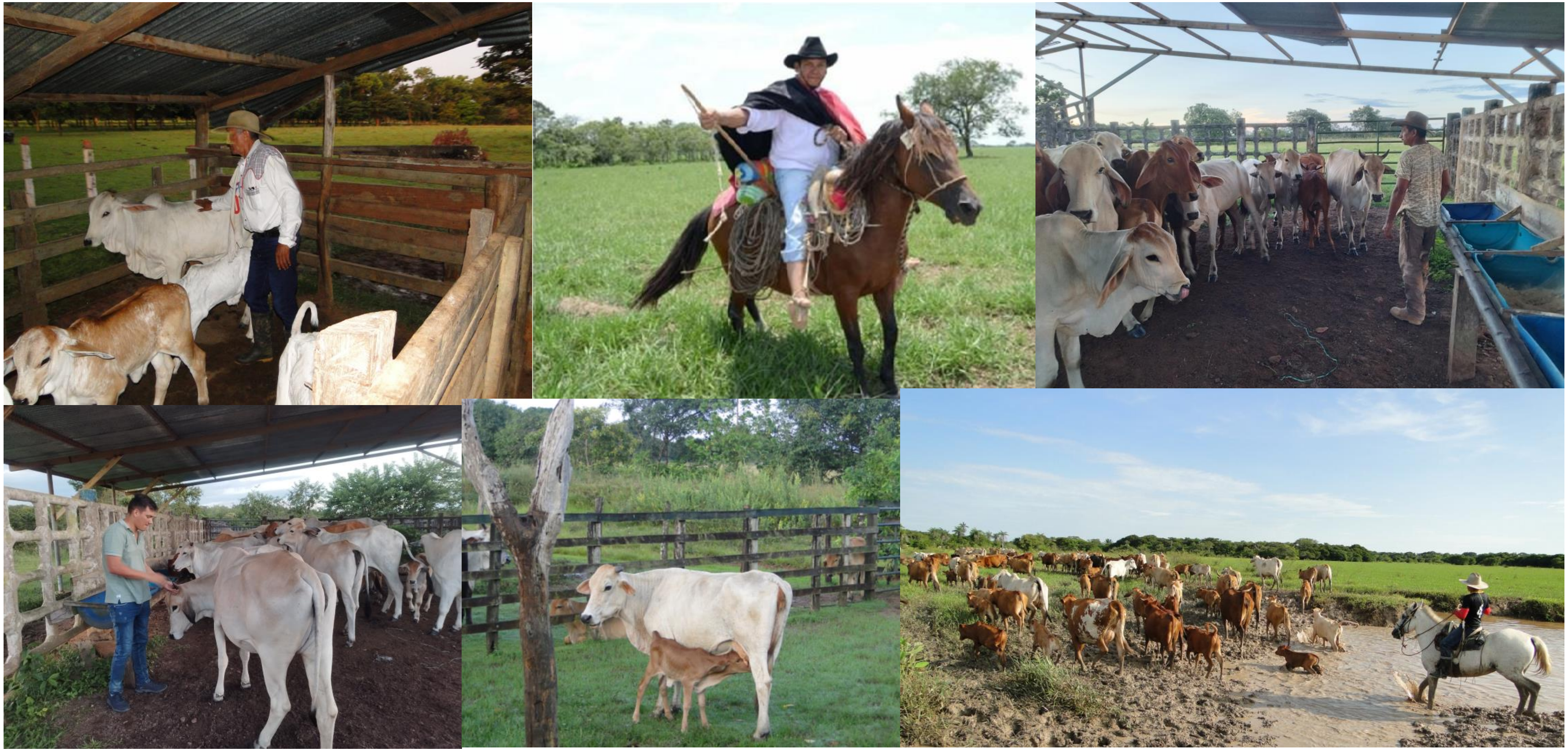

Figure S2. Farmer-livestock interaction, Vichada, Colombia.
